# The ethylene response factor Pti5 contributes to potato aphid resistance in tomato independent of ethylene signalling

**DOI:** 10.1093/jxb/eru472

**Published:** 2014-12-11

**Authors:** Chengjun Wu, Carlos A. Avila, Fiona L. Goggin

**Affiliations:** ^1^Department of Entomology, University of Arkansas, Fayetteville, AR 72701, USA; ^2^Department of Horticultural Sciences, Texas A&M AgriLife Research, Weslaco, TX 78596, USA

**Keywords:** Basal resistance, ERF, EREBP, insect resistance, *Macrosiphum euphorbiae*, *Mi-1*.2.

## Abstract

Ethylene response factors (ERFs) comprise a large family of transcription factors that regulate numerous biological processes including growth, development, and response to environmental stresses. Here, we report that *Pti5*, an ERF in tomato [*Solanum lycopersicum* (Linnaeus)] was transcriptionally upregulated in response to the potato aphid *Macrosiphum euphorbiae* (Thomas), and contributed to plant defences that limited the population growth of this phloem-feeding insect. Virus-induced gene silencing of *Pti5* enhanced aphid population growth on tomato, both on an aphid-susceptible cultivar and on a near-isogenic genotype that carried the *Mi-1.2* resistance (R) gene. These results indicate that *Pti5* contributes to basal resistance in susceptible plants and also can synergize with other R gene-mediated defences to limit aphid survival and reproduction. Although *Pti5* contains the ERF motif, induction of this gene by aphids was independent of ethylene, since the ACC deaminase (*ACD*) transgene, which inhibits ethylene synthesis, did not diminish the responsiveness of *Pti5* to aphid infestation. Furthermore, experiments with inhibitors of ethylene synthesis revealed that *Pti5* and ethylene have distinctly different roles in plant responses to aphids. Whereas *Pti5* contributed to antibiotic plant defences that limited aphid survival and reproduction on both resistant (*Mi-1.2+*) and susceptible (*Mi-1.2–*) genotypes, ethylene signalling promoted aphid infestation on susceptible plants but contributed to antixenotic defences that deterred the early stages of aphid host selection on resistant plants. These findings suggest that the antixenotic defences that inhibit aphid settling and the antibiotic defences that depress fecundity and promote mortality are regulated through different signalling pathways.

## Highlight

This study demonstrated that the ethylene response factor Pti5 contributes to antibiotic defences against aphids, whereas ethylene contributes to antixenotic but not antibiotic aphid resistance.

## Introduction

Aphids are a large and economically damaging group of insects that are specialized to feed on phloem sap and that can reduce crop yields by withdrawing photoassimilates, manipulating plant growth and resource allocation, and transmitting phytopathogenic viruses (Goggin, 2007). Plants limit losses to aphids through a variety of defences, including sensory cues that deter aphids from settling on the plant (i.e. antixenosis), and toxins or other resistance factors that suppress aphid fecundity and promote mortality (i.e. antibiosis) (Smith and Chuang, 2014). In some cases, host plant resistance depends upon single dominant resistance genes (so-called R genes) that act against specific pest biotypes, resulting in an incompatible interaction. For example, the *Mi-1.2* gene in tomato [*Solanum lycopersicum* (Linnaeus)], blocks infestation by some but not all populations of the potato aphid, *Macrosiphum euphorbiae* (Thomas) (Rossi *et al.*, 1998; Goggin *et al.*, 2001). However, even in compatible interactions in which relevant R genes are absent and aphids can colonize the host, plants deploy defences that reduce the magnitude of infestations. These defences, present in compatible or ‘susceptible’ hosts, are described as basal resistance and provide a foundation upon which R gene-mediated resistance builds. Basal resistance encompasses not only direct defences with toxic or repellent effects on the pest but also any plant traits that intercept the chain of events leading to susceptibility (Dangl and Jones, 2001; de Wit, 2007; Niks and Marcel, 2009; Anderson *et al.*, 2014). Both basal and R gene-mediated resistance involve induced responses including hormone signalling and transcriptional reprogramming, and there is often extensive overlap in the signalling networks and transcription factors that contribute to these two forms of defence (Navarro *et al.*, 2004).

This study explored the role of the Pti5 transcription factor in basal and *Mi-1.2-*mediated aphid resistance tomato, as well as the potential interactions of this ethylene response factor (ERF) with the hormone ethylene. Ethylene is a gaseous hormone that regulates plant responses to numerous biotic and abiotic stimuli including mechanical wounding, chewing insects, and pathogen infection (O’Donnell *et al.*, 1996; Knoester *et al.*, 1998; Solano and Ecker, 1998; von Dahl and Baldwin, 2007; Mantelin *et al.*, 2009). Ethylene signalling activates the transcription of numerous pathogenesis-related (PR) proteins and other genes that contribute to defence, and many of these ethylene-inducible genes contain a conserved 11bp promoter sequence (TAAGAGCCGCC) called the GCC box (Eyal *et al.*, 1993). ERFs, formerly known as ethylene-responsive element binding proteins (EREBPs), are transcription factors that specifically bind to genes having the GCC box. The ERF family has important roles in regulating biological processes related to plant growth, development, metabolism, and response to biotic and abiotic stresses (Ohme-Takagi and Shinshi, 1995; Stockinger *et al.*, 1997; Liu *et al.*, 1998; Yamamoto *et al.*, 1999; Gu *et al.*, 2000; van der Fits and Memelink, 2000; Berrocal-Lobo *et al.*, 2002; Nakano *et al.*, 2006). Ethylene may regulate PR gene expression through ERF transcription factors (Eyal *et al.*, 1993; Ohme-Takagi and Shinshi, 1995; Solano *et al.*, 1998), and ERFs can act as either transcriptional activators or repressors of GCC box-mediated gene expression (Fujimoto *et al.*, 2000; Yang *et al.*, 2005).

ERFs have been characterized in several model plants including *Arabidopsis thaliana* (Büttner and Singh, 1997; Solano *et al.*, 1998; Fujimoto *et al.*, 2000; Nakano *et al.*, 2006), tomato (Zhou *et al.*, 1997), *Oryza sativa* (Nakano *et al.*, 2006), and *Nicotiana tobaccum* (Ohme-Takagi and Shinshi, 1995; Suzuki *et al.*, 1998). In tomato, the Pti5 transcription factor containing the ERF binding domain was isolated based on its physical interaction with a serine–threonine kinase encoded by the *Pto* gene (*Pti*=*Pto* interacting) (Zhou *et al.*, 1997). *Pto* acts in conjunction with the *Prf* R gene to confer resistance against certain races of the bacterial pathogen *Pseudomonas syringae* pv. *tomato* (Salmeron *et al.*, 1996; Pedley and Martin, 2003). *Pti5* acts downstream of *Pto* and *Prf* to upregulate expression of defence genes including the GCC box-containing PR genes encoding β-1,3-glucanase and osmotin (He *et al.*, 2001). Furthermore, overexpression of *Pti5* in a susceptible tomato background enhances basal resistance to *P. syringae* pv. *tomato*, indicating that *Pti5* contributes to pathogen resistance (He *et al.*, 2001). Analysis of transcriptional responses in melon (*Cucumis melo*) to infestation by the cotton-melon aphid (*Aphis gossypii*) also indicates that aphid feeding upregulates ERFs, and that the *Vat* gene for aphid resistance may enhance this response (Anstead *et al.*, 2010). Thus, ERFs could potentially play a role in R gene-mediated aphid resistance.

While the impact of ERFs on aphid resistance has not been tested previously, several earlier studies have examined the role of ethylene in plant–aphid interactions, and aphids have been shown to induce an ethylene burst in several different plant species (Dillwith *et al.*, 1991; Anderson and Peters, 1994; Miller *et al.*, 1994; Argandona *et al.*, 2001; Mantelin *et al.*, 2009). In barley, this ethylene burst was reported to be greater in an aphid-resistant genotype than in a susceptible cultivar, suggesting that ethylene may be associated with resistance (Argandona *et al.*, 2001). However, the opposite trend was observed in alfalfa plants challenged with the spotted alfalfa aphid (*Therioaphis maculata*) and wheat plants challenged with the Russian wheat aphid (*Diuraphis noxia*); in these cases, ethylene generation in response to aphids was greater in susceptible genotypes than in resistant or tolerant lines (Dillwith *et al.*, 1991; Miller *et al.*, 1994). In tomato, artificially exposing plants to ethylene also rendered them more susceptible to the green peach aphid, *Myzus persicae* (Boughton *et al.*, 2006). Furthermore, Mantelin *et al.* (2009) found that inhibiting ethylene synthesis in tomato reduced host suitability for the potato aphid on a susceptible genotype but had no effect on a resistant tomato genotype that carries the *Mi-1.2* R gene. These results indicate that ethylene signalling in response to aphids can actually facilitate the infestation process in some cases, but that the effects of ethylene vary among genotypes.

In this study, we investigated the role of the ERF *Pti5* in plant–aphid interactions in resistant (*Mi-1.2*+) and susceptible (*Mi-1.2*–) tomato genotypes, and compared its effects with those of ethylene. We demonstrated that the potato aphid causes transcriptional upregulation of *Pti5*, and that this gene contributes to antibiotic defences against aphids in both susceptible and resistant plants. Ethylene was not required for upregulation of *Pti5* by aphids and did not contribute to antibiosis in either genotype. Therefore, we concluded that *Pti5* contributes to potato aphid resistance in tomato independent of ethylene signalling. Surprisingly, we also discovered that, even though ethylene promoted infestations on susceptible tomato plants, it contributed to the early stages of antixenotic defences in plants that carried the *Mi-1.2* gene for aphid resistance. These results indicate that antibiosis and antixenosis depend upon different signalling pathways and further our understanding of the molecular basis of basal and R gene-mediated aphid resistance.

## Materials and methods

### Plant and insect materials

The following tomato (*S. lycopersicum* L.) genotypes were used in this study: three aphid-susceptible cultivars that lack the *Mi-1.2* resistance gene (‘MoneyMaker’, ‘UC82B’, and ‘Castlemart’); a transgenic line (143-25) that was generated by transforming ‘MoneyMaker’ with *Mi-1.2* (Milligan *et al.*, 1998); a cultivar, ‘Motelle’, that is near-isogenic to ‘MoneyMaker’ but that carries a 650kb introgressed region containing *Mi-1.2* (Kaloshian *et al.*, 1998); a transgenic line, *ACD*, that was generated by transforming the cultivar ‘UC82B’ with a gene from *Pseudomonas chloroaphis* that encodes 1-aminocyclopropane-1-carboxylic acid (ACC) deaminase (ACD; Klee *et al.*, 1991); two mutant tomato lines, *acyl-CoA oxidase 1A* (*acx1*) and *suppressor of prosystemin-mediated responses2* (*spr2*), that were generated in the ‘Castlemart’ genetic background (Li *et al.*, 2003); and a transgenic line that was generated in a ‘MoneyMaker’ background and that expresses the naphthalene/salicylate hydroxylase (*NahG*) transgene from *Pseudomonas putida* (Gaffney *et al.*, 1993). The aphid-resistant (*Mi-1.2*+) transgenic line 143-25 was used in initial gene expression experiments because it provides an isogenic control to the susceptible (*Mi-1.2*–) line ‘MoneyMaker’. However, for subsequent bioassays, ‘Motelle’ (*Mi-1.2*+) was utilized instead of 143-25 because levels of *Mi-1.2*-mediated resistance appear to decline as the *Mi-1.2* transgene is passed through more than one generation (Goggin *et al.*, 2004).

Tomato plants were grown in 0.2-l pots using LC1 Sunshine potting mix (Sungro Horticulture, MI, USA) supplemented with 15-9-12 Osmocote Plus slow-release fertilizer (Scotts-MiracleGro Company, OH, USA). Seedlings were grown under uniform greenhouse conditions (24–27 °C and a 16:8h light:dark photoperiod) until imposition of experimental treatments. Plants were watered with a dilute nutrient solution containing 1000 ppm CaNO_3_ (Hydro Agri North America, FL, USA), 500 ppm MgSO_4_ (Giles Chemical Corp, NC, USA), and 500 ppm 4-18-38 Gromore fertilizer (Gromore, CA, USA). The potato aphid (*M. euphorbiae*, isolate WU11) was reared on a combination of tomato (cv. ‘UC82’), potato (*Solanum tuberosum* L.), and jimson weed (*Datura stramonium* L.) plants at 23 °C with a 16h light photoperiod.

### Genetic crosses and genotype selection

The cultivar ‘Motelle’ (MT) and the transgenic line *ACD* were crossed to produce MT×*ACD* plants carrying both the *Mi-1.2* gene and the *ACD* gene. The (MT×*ACD*) F_1_ generation was PCR screened for the presence of both genes using the following sets of primers: *ACD* forward, 5ʹ-CGATACGCTGGTTTCCATC-3ʹ, and reverse, 5ʹ-CGTCCTCTTCCGTAATCTCG-3ʹ; and *Mi-1.2* forward, 5ʹ-CTAGAAAGTCTGTTTGTGTCTAACAAAGG-3ʹ, and reverse, 5ʹ-CTAAGAGGAATCTCATCACAGG-3ʹ. Similar to *Mi-1.2*, the *ACD* transgene behaves as a single dominant gene inherited in a Mendelian fashion when transgenic tomato plants are crossed with other tomato lines (Reed *et al.*, 1995); therefore, heterozygous F_1_ plants were used for subsequent assays.

### Gene expression analysis

In order to assess the effects of aphid feeding on gene expression in plants with and without the *Mi-1.2* resistance gene, ‘MoneyMaker’ and 143-25 were mock infested with empty nylon sleeve cages that each covered a single leaflet (control), or infested with potato aphids (25 aphids per sleeve cage; two sleeve cages per plant; three plants per treatment group). At 6 and 24h after infestation (HAI), the aphids were gently removed with a paintbrush, and the leaf tissue was flash frozen with liquid nitrogen and stored at –80 °C. Total RNA was extracted from each sample using TRI Reagent (Molecular Research Center, OH, USA), and then was DNase treated with TURBO DNA-free (Ambion, Austin, TX, USA) followed by reverse transcription of 0.5 µg of RNA using Superscript II reverse transcriptase (Invitrogen, Carlsbad, CA, USA) in a 20 µl reaction volume. The following set of primers were then used for quantitative PCR (qPCR): *Pti5* (GenBank accession no. U89256) forward, 5ʹ-GCGAGGTGCTAAGGCACTAC-3ʹ, and reverse, 5ʹ-TGCCA AGAAATTCTCCATGC-3ʹ; *E4* (GenBank accession no. S44989.1) forward, 5ʹ-TGATGCTCAGGCTCAACTGG-3ʹ, and reverse, 5ʹ-ACTGCTTACAACCTCTGCCC-3ʹ; *RPL2* (GenBank accession no. X64562) forward, 5ʹ-GAGGGCGTACTGAGAAACCA-3ʹ, and reverse, 5ʹ-CTTTTGTCCAGGAGGTGCAT-3ʹ. Real-time (RT)-qPCR was performed with a QuantiTect SYBR Green PCR kit (Qiagen, CA, USA) in a 20 μl reaction. The StepOnePlus^TM^ Real-Time PCR system (Applied Biosystems) was used for fluorescence detection. Two technical replicates per biological sample were run. The PCR conditions were as follows: 15min of activation at 95 °C, 40 amplification cycles (94 °C for 15 s, 55 °C for 30 s, and 72 °C for 30 s, with data acquisition at the end of each cycle), and a final data acquisition step to generate melting curves from 65 to 95 °C every 0.3 °C. Melting curves were performed for all samples to verify single amplification product. For each primer set, the amplification efficiency was calculated using the formula E=10^(–1/Ct slope)^ from data generated from serial dilutions of a set of bulked cDNA standards having equal aliquots from all the samples (Rasmussen, 2000). Relative gene expression was calculated using Pfaffl methodology (Pfaffl, 2001). Data were normalized to the expression levels of the endogenous control ribosomal protein L2 gene (*RPL2*), and gene expression for each treatment group was calculated relative to the wild-type (WT) control group in each experiment. For statistical analysis, the relative expression values for each treatment group were log_2_ transformed to stabilize variances. Data were analysed by two-way analysis of variance (ANOVA) and means for significant effects at α=0.05 were separated using Student’s *t*-test with JMP^®^ Pro 9.0 (SAS Institute, NC, USA).

### Aphid bioassays

#### No-choice tests 

Aphids were restricted to single leaflets using lightweight mesh clip cages (~3.5cm diameter). Six-week old tomato plants were infested with four to five apterous young adult potato aphids per cage, with at least two cages per plant, and maintained at 20 °C and with a 16:8h light:dark photoperiod. At 5–7 days after infestation (DAI), the total number of live aphids (adults and nymphs) was recorded in each cage. Each plant was treated as a biological replication, and separate cages were treated as subsamples. The average number of aphids per cage per plant was analysed by one- or two-way ANOVA, depending upon whether there were one or two independent variables in the experiment. If the ANOVAs revealed statistically significant main effects or significant interactions between independent variables, mean separations were performed using Student’s *t*-test to identify differences among treatment groups (α=0.05). All statistics were performed with JMP^®^ Pro 9.0.

#### Choice tests 

Eight-week-old ‘MoneyMaker’ and ‘Motelle’ plants were sprayed with 0.01% Tween 20 with or without 1mM aminoethoxyvinylglycine (AVG; Valent USA Corporation, IL, USA). Six hours after treatment, when the foliage was fully dry, the plants were arranged in pairs on laboratory benches. For each possible pairing of plants, there were six replicate pairs per experiment. A choice arena consisting of a pedestal with a styrofoam platform approximately 10cm in diameter was placed half way between the plant pairs as described by Avila *et al.* (2012). Each arena was in contact with one terminal leaflet of a mature leaf on each of the two plants in the pair. The leaf positions 6–10 (first true leaf=1) were used for this assay. Each leaflet used for the choice test was isolated from the rest of the plant using a circular plastic shield (11cm in diameter) with Tanglefoot Pest Barrier (The Tanglefoot Company, MI, USA) around the edge. Twenty adult apterous potato aphids were released onto the centre of each platform at the start of the experiment. The number of aphids and offspring were counted on each leaflet at four time points after release (1, 6, 24, and 48 HAI). The data for each comparison were analysed by a matched-pairs one-sided *t*-test within each time point in JMP^®^ Pro 9.0.

### Silencing of *Pti5*

#### Construction of TRV-*Pti5*

Virus-induced gene silencing (VIGS) was performed to suppress expression of *Pti5* in tomato using the tobacco rattle virus (TRV) system. VIGS was performed as described previously (Wu *et al.*, 2011; Avila *et al*., 2012, 2013). The TRV-*Pti5* construct consisted of a 302bp fragment corresponding to nt 379–680 of the tomato *Pti5* gene (GenBank accession no. U89256), which was amplified using the following primers: forward, 5ʹ- CGGTCTAGAAGTGAACGCCTCTGTTTCAG-3ʹ and reverse 5ʹ-CGGGGATCCGTCGCTGCAAACAATTCCAT-3ʹ. The *Pti5* fragment was cloned into the pYL156 vector to generate the TRV-*Pti5* silencing construct. The pYL156-*Pti5* vector was transformed into *Escherichia coli* strain DH10B, and the insert was confirmed by sequencing and then introduced into *Agrobacterium tumefaciens* strain GV3101. For the empty control vector (TRV-CV), we used pYL156 carrying a 396bp insert from the β-glucuronidase reporter gene (GenBank accession no. S69414.1) to achieve a control vector that would have similar movement and proliferation in the plant compared with the experimental vector (Wu *et al.*, 2011). Additionally, a construct that silences the phytoene desaturase gene (*PDS*, GenBank accession no. M88683.1) (Wu *et al.*, 2011) was used in a separate set of plants as a visual reporter to monitor the onset of VIGS.

#### Agro-infiltration and bioassays 

The TRV vectors were infiltrated into tomato as described previously (Wu *et al.*, 2011). Briefly, 2-week-old tomato plants were vacuum infiltrated with TRV-*Pti5*, TRV-CV, or TRV-*PDS* and kept at 20 °C in a growth chamber with a 16:8h light:dark photoperiod until ready for bioassay. No-choice aphid bioassays were performed as described above when monitor plants infiltrated with the TRV-*PDS* vector showed a widespread uniform bleaching phenotype (~4 weeks after infiltration; inoculum level of five aphids per cage, four cages per plant; the number of live aphids per cage scored at 4 and 7 DAI).

## Results

### Aphid infestation induces *Pti5* expression in tomato

**Fig. 1.**
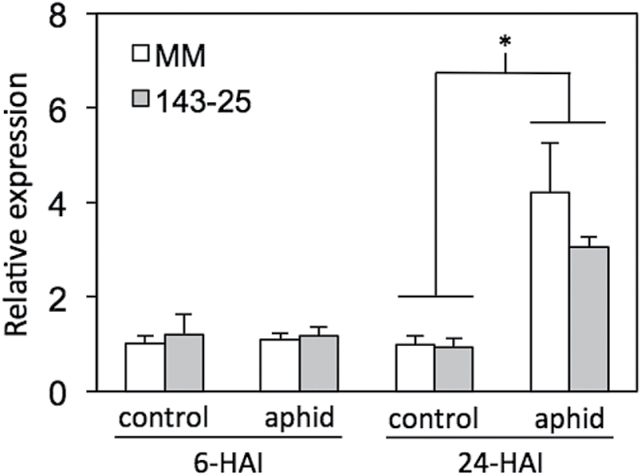
Tomato *Pti5* gene expression in response to aphid infestation. The aphid-susceptible tomato cv. ‘MoneyMaker’ (MM) and a transgenic line transformed with the *Mi-1.2* aphid resistance gene (143-25) were mock infested with empty cages (control) or infested with aphids (25 aphids per sleeve cage; two sleeve cages per plant). Relative gene expression was measured by RT-qPCR at 6 and 24 HAI. Expression was normalized relative to the endogenous control *RPL2* gene. Asterisks (*) denote statistically significant differences at α=0.05 [*n*=3; error bars represent standard error of the mean (SEM)].

Gene expression of *Pti5* was monitored by RT-qPCR at 6 and 24h HAI in the aphid-susceptible tomato cultivar ‘MoneyMaker’ and the aphid-resistant transgenic line 143-25, which carries the *Mi-1.2* transgene in a ‘Moneymaker’ genetic background (Fig. 1). No change in gene expression was observed at 6 HAI, but at 24 HAI, *Pti5* transcripts were 3- to 4-fold higher in infested plants than in uninfested controls for both genotypes (*P*<0.05). No difference in *Pti5* transcript abundance was observed between ‘MoneyMaker’ and 143-25 (*P*=0.7697), which suggested that *Mi-1.2* does not influence constitutive or aphid-responsive levels of *Pti5* expression.

### The *Pti5* gene contributes to antibiotic defences against aphids in both resistant and susceptible tomato genotypes

**Fig. 2.**
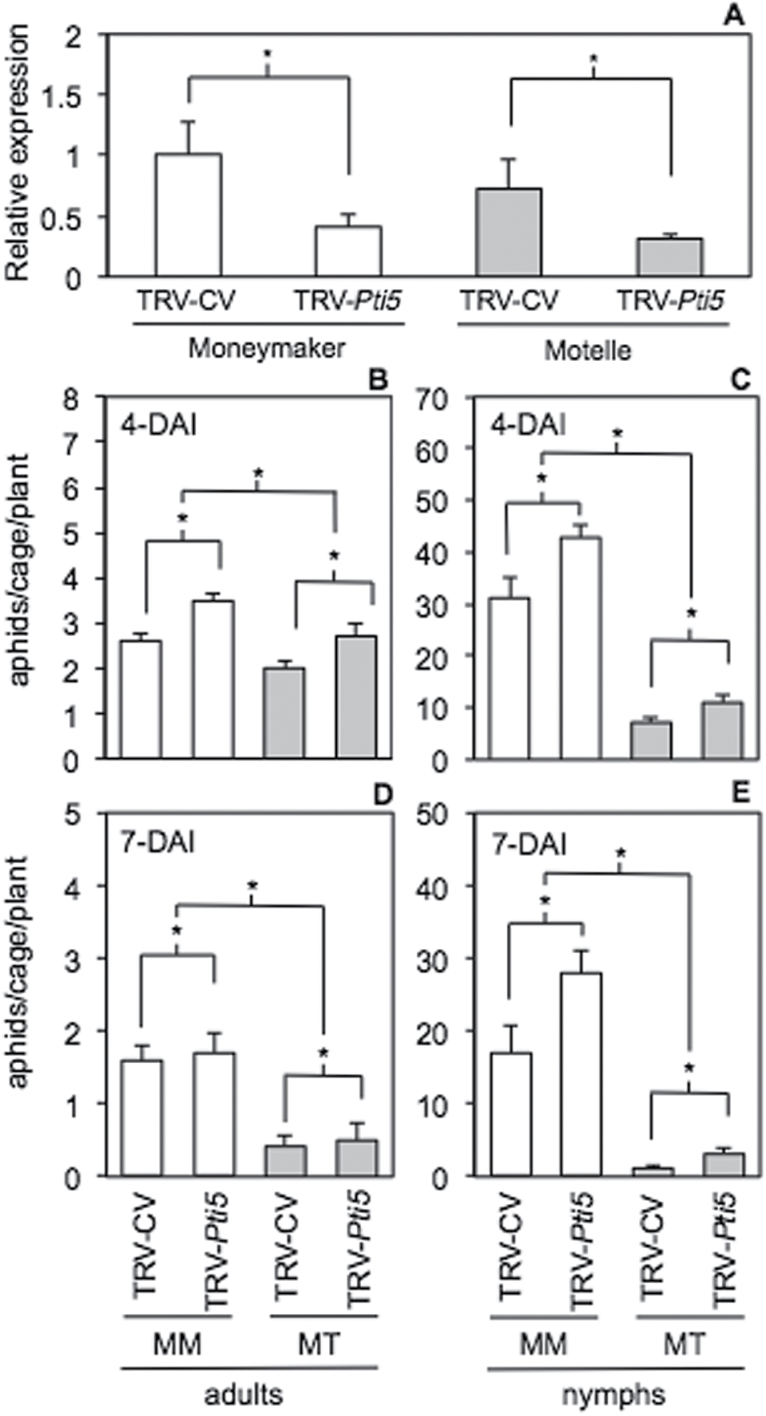
Aphid population growth on *Pti5*-silenced tomato plants. The aphid-susceptible tomato cultivar ‘MoneyMaker’ (MM) and the near-isogenic line ‘Motelle’ (MT) carrying the *Mi-1.2* resistance gene were treated with a TRV vector modified to suppress expression of *Pti5* (TRV-*Pti5*) or with a control vector of comparable size that does not silence any endogenous genes in tomato (TRV-CV). The silencing efficiency for TRV-*Pti5* was corroborated by RT-qPCR normalized relative to the endogenous *RPL2* gene (A). Plants were infested with aphids confined to clip cages (five young adult potato aphids per cage; five cages per plant; eight plants per treatment group), and the number of live adults (B, D) and offspring (C, E) were recorded at 4 and 7 DAI. Values for *Pti5* expression (A) and aphid survival and reproduction (B–E) were analysed by two-way ANOVA and means separated by Student’s *t*-test. Asterisks (*) denote statistically significant differences at α=0.05, and error bars represent SEM (*n*=3 for A, *n*=8 for B–E).

To investigate if *Pti5* influenced plant defences against aphids, VIGS was performed to suppress *Pti5* expression in the aphid-susceptible tomato ‘Moneymaker’ and the near-isogenic resistant line ‘Motelle’ carrying the *Mi-1.2* gene. Plants were infiltrated either with a construct designed to silence *Pti5* (TRV-*Pti5*) or with a control vector of comparable size (TRV-CV). Compared with plants that received the vector control, plants infiltrated with TRV-*Pti5* had significantly lower abundance of *Pti5* transcripts in the foliage (Fig. 2A; *P*=0.0013), and the efficacy of silencing was similar in the two genotypes. To measure antibiosis, plants were inoculated with caged adult aphids in a no-choice test, and insect survivorship and reproduction was monitored at 4 and 7 DAI. The aphid-susceptible cultivar ‘MoneyMaker’ had higher numbers of live adults (Fig. 2B; *P*=0.0098) and offspring (Fig. 2C; *P*<0.0001) than the aphid-resistant genotype ‘Motelle’ at 4 DAI. Regardless of the genotype tested, plants treated with TRV-*Pti5* also supported higher numbers of surviving adults (Fig. 2B; *P*=0.0033) and live offspring (Fig. 2C; *P*=0.0063) than the TRV-CV-treated plants. The percentage increase in surviving adults (35% on ‘MoneyMaker’ and 35% on ‘Motelle’) and offspring (37% on ‘MoneyMaker’ and 40% on ‘Motelle’) that resulted from silencing of *Pti5* was similar on the two genotypes, and there was no statistically significant interaction between VIGS treatment and plant genotype (*P*>0.1). This suggested that the effects of silencing by TRV-*Pti5* was similar in the two genetic backgrounds. The same patterns of aphid population growth observed at 4- DAI persisted at 7 DAI (Fig. 2D, E), indicating that the effect of *Pti5* silencing on aphid population growth remained stable for the time period tested.

### In contrast to *Pti5*, ethylene signalling depresses basal resistance in susceptible tomato and does not contribute to antibiosis in resistant plants

**Fig. 3.**
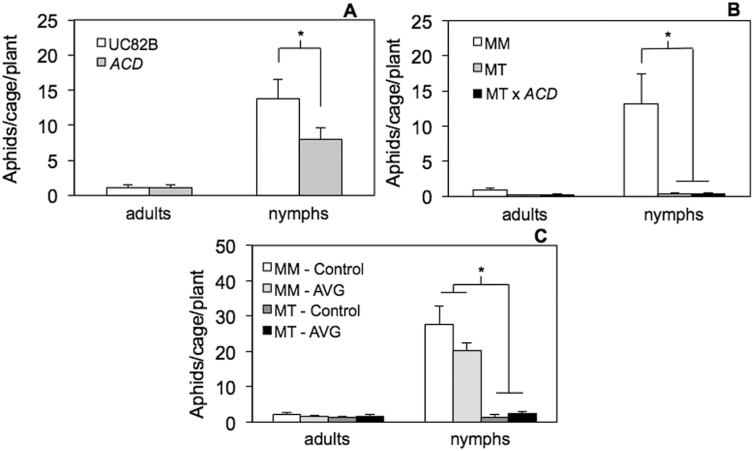
Aphid population growth on tomato plants with compromised ethylene signalling. Aphid survival and reproduction was compared in no-choice tests on an *ACD* transgenic line deficient in ethylene synthesis and the WT background for this line, ‘UC82B’ (A); and on hybrids between *ACD* and the resistant cultivar ‘Motelle’ (MT×*ACD*) (B). Additionally, aphid survival and reproduction was measured on resistant ‘Motelle’ (MT) and susceptible ‘MoneyMaker’ (MM) plants treated with AVG or a blank carrier solution (control). The numbers of adult aphids and their offspring (nymphs) were analysed by two-way ANOVA and means separated by Student’s *t*-test. Asterisks (*) denote statistically significant differences at α=0.05, and error bars represent SEM (*n*=14 for A and *n*=6 for B and C).

Because *Pti5* is classified as an ERF, we also investigated the influence of ethylene on defences that affect herbivore survival and reproduction (i.e. antibiosis) using the *ACD* transgene. Plants that express ACD are impaired in ethylene synthesis because this enzyme degrades ACC, an essential precursor for ethylene synthesis (Klee *et al.*, 1991). We performed no-choice tests to compare the survival and offspring production of adult aphids on the transgenic line *ACD* (which carries the *ACD* gene in an aphid-susceptible ‘UC82B’ background), hybrids that carry both the *ACD* transgene and the *Mi-1.2* resistance gene (MT×*ACD*), and resistant (‘Motelle’) and susceptible (‘UC82B’, ‘Moneymaker’) cultivars with WT ethylene signalling. At 7 DAI, the average number of aphid offspring (i.e. nymphs) was ~40% lower on the *ACD* transgenic line than on the WT background ‘UC82B’ (Fig. 3A, *P*=0.0450). Since *ACD* did not affect adult survival (Fig. 3A, *P*=0.5000), the decrease in reproduction on *ACD* was probably due to a decrease in adult fecundity. In contrast, *ACD* had no significant impact on offspring production (*P*=0.4326) or adult aphid survival (*P*=0.3226) in a genetic background that carried the *Mi-1.2* resistance gene (Fig. 3B). A similar trend was observed when we used a pharmacological approach to disrupt ethylene synthesis in ‘MoneyMaker’ and ‘Motelle’ by applying AVG to block the activity of 1-aminocyclopropane-1-carboxylate synthase, which generates a necessary precursor for ethylene synthesis (Yu and Yang, 1979; Huai *et al.*, 2001). AVG treatment lowered offspring production on the susceptible genotype ‘MoneyMaker’ but not on the resistant isoline ‘Motelle’, and as denoted by the statistically significant interaction between AVG treatment and plant genotype (Fig. 3C; two-way ANOVA, main effect of AVG treatment: *P*=0.4889; main effect of genotype: *P*<0.0001; and the interaction effect between AVG treatment and genotype: *P*=0.0401). AVG did not impact adult survival on either genotype (*P*>0.10). These results indicated that ethylene signalling promotes aphid infestation on susceptible tomato genotypes but not on resistant genotypes that carry the *Mi-1.2* resistance gene.

### Ethylene signalling promotes aphid settling on susceptible plants but may contribute to antixenosis in plants that carry *Mi-1.2*

**Fig. 4.**
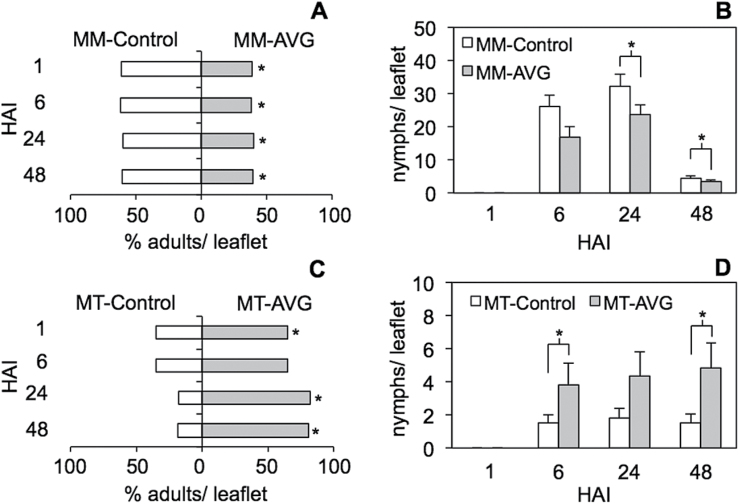
Aphid host preference between plants with and without an inhibitor of ethylene synthesis. The aphid-susceptible cultivar ‘MoneyMaker’ (MM) and the near-isogenic resistant line ‘Motelle’ (MT) were treated with an inhibitor of ethylene synthesis (AVG) or mock treated with solvent (control). To determine if aphids had a preference between AVG-treated or mock-treated plants, adult aphids were placed on choice arenas between paired plants and allowed to move back and forth between the plants (20 aphid per /area; six arenas per comparison). The percentage of adults that were located on treated plants versus controls (A, C) was recorded at 1, 6, 24, and 48 HAI, along with the number of offspring produced on each treatment group (B, D). Comparisons labelled with an asterisk (*) were statistically different at α=0.05 according to one-sided matched-pair comparisons. Error bars in (B) and (C) represent SEM.

We also tested the effect of ethylene on the plant’s ability to deter aphid settling during the host selection process (i.e. antixenosis or non-preference). To avoid the potential effects of multiple loci that are segregating in the ‘MT×*ACD*’ hybrids, we chose instead to manipulate ethylene signalling by applying AVG or a blank carrier solution to the near-isolines ‘MoneyMaker’ and ‘Motelle’. Aphid settling was tested in choice tests in which groups of 20 adult aphids were placed on platforms (choice arenas) between paired plants with and without AVG treatment. Aphids typically moved off the arena onto the foliage within minutes of introduction, and were free to move back and forth between the two plants by crossing the arena. At 1, 6, 24, and 48 HAI, we recorded the position of adults, as well their offspring production, which is a well-established marker of host plant acceptance (Powell *et al.*, 2006). On the susceptible cultivar ‘MoneyMaker’, aphids had a significant preference to settle on plants treated with the control solution compared with plants treated with AVG (Fig. 4A, *P*<0.05 at all time points), and by 24 HAI, reproduction was also higher on the controls (Fig. 4B; matched-pairs test, 6 HAI: *P*=0.1146; 24 HAI: *P*=0.0304; and 48 HAI: *P*=0.0350. No nymphs were observed at 1 HAI). In contrast, on the resistant line ‘Motelle’, aphids preferred to settle (Fig. 4C) and reproduce (Fig. 4D) on plants treated with AVG than on plants treated with the blank carrier solution (adult settling: matched-pairs test, 1 HAI: *P*=0.0343; 6 HAI: *P*=0.1054; 24 HAI: *P*=0.0189; and 48 HAI: *P*=0.0272; offspring production: matched-pairs test, 6 HAI: *P*=0.0421; 24 HAI: *P*=0.0654; and 48 HAI: *P*=0.0351). These results indicated that ethylene signalling promotes aphid host acceptance on susceptible plants, but contributes to antixenosis (i.e. deterrent defences) in plants that carry the *Mi1.2* resistance gene.

**Fig. 5.**
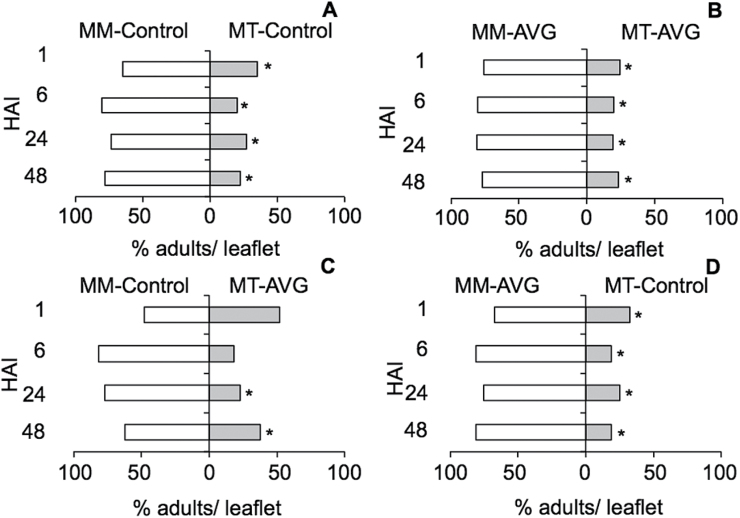
Aphid host preference between plants with and without *Mi-1.2*, in the presence or absence of an inhibitor of ethylene synthesis. The aphid-susceptible cultivar ‘MoneyMaker’ (MM) and the near-isogenic resistant line ‘Motelle’ (MT) were treated with AVG or mock treated with solvent (control), and choice assays were performed to determine whether AVG influenced the aphids’ ability to discriminate between the two genotypes (20 aphids per area; six arenas per comparison). The percentage of adults that were located on treated plants versus controls was recorded at 1, 6, 24, and 48 HAI. Comparisons labelled with an asterisk (*) were statistically different at α=0.05 according to one-sided matched-pair comparisons.

We also performed additional choice tests to investigate whether ethylene influenced aphid host preferences between genotypes. When aphids were offered a choice between ‘MoneyMaker’ plants treated with AVG or ‘Motelle’ plants treated with AVG, they consistently preferred ‘MoneyMaker’ over ‘Motelle’ (Fig. 5B, *P*<0.05 at all time points tested), indicating that inhibiting ethylene synthesis did not eliminate antixenosis in the resistant line. However, when aphids were offered a choice between AVG-treated ‘Motelle’ and mock-treated ‘MoneyMaker,’ AVG appeared to delay the aphids’ ability to discriminate between the resistant and susceptible genotypes (Fig. 5C). Whereas aphid numbers were significantly higher on mock-treated ‘MoneyMaker’ compared with mock-treated ‘Motelle’ as early as 1 HAI (Fig. 5A, *P*<0.05 at all time points), aphids presented with ‘MoneyMaker’ controls and AVG-treated ‘Motelle’ plants did not show a statistically significant preference for ‘MoneyMaker’ until 24 HAI (Fig. 5C; 1 HAI, *P*=0.6222; 6 HAI, *P*=0.0744; 24 HAI *P*=0.0014; and 48 HAI, *P*=0.0356). In contrast, applying AVG to ‘MoneyMaker’ plants only (MM-AVG) did not inhibit aphids from selecting ‘MoneyMaker’ over mock-treated ‘Motelle’ plants (Fig. 5D; *P*< 0.01 at all time points). Together, these results suggested that, in resistant plants, ethylene signalling may contribute to antixenotic defences that act early (<24h) in the plant–aphid interaction.

### Induction of *Pti5* expression by aphids is independent of ethylene, jasmonic acid (JA), or salicylic acid (SA) accumulation

**Fig. 6.**
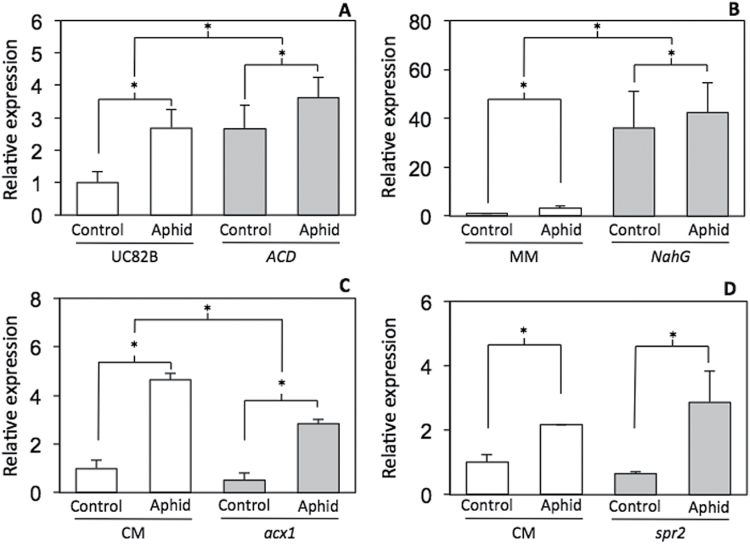
Role of plant defensive hormones in aphid-induced *Pti5* gene expression. Relative expression of *Pti5* was measured by RT-qPCR 48h after potato aphid infestation in the following tomato hormone-deficient plants: ethylene-deficient *ACD* and untransformed control ‘UC82B’ (A); SA-deficient *NahG* and untransformed control ‘MoneyMaker’ (MM) (B); and JA-deficient *acx1* (C) and *spr2* (D) with their WT background ‘Castlemart’ (CM). Gene expression was normalized relative to the *RPL2* gene. Asterisks (*) denote statistically significant differences at α=0.05, and error bars represent SEM (*n*=6 for A, *n*=4 for B and C, and *n*=3 for D).

Although several studies on ERF-like genes encoding GCC box-binding factors have focused on their role in ethylene signalling (Solano and Ecker, 1998; Suzuki *et al.*, 1998), our results indicated that *Pti5* and ethylene signalling have different roles in plant–aphid interactions. To further corroborate that induction of *Pti5* acted independent of ethylene signalling, we monitored *Pti5* gene expression at 48 HAI in the ethylene-deficient *ACD* transgenic line. *Pti5* gene expression was induced by aphids to a similar extent in both *ACD* and the WT control ‘UC82B’, indicating that aphid induction was independent of ethylene levels. However, basal levels of the *Pti5* transcript in uninfested plants were higher in *ACD* than in ‘UC82B’, suggesting that constitutive expression of *Pti5* may be inhibited by ethylene (Fig. 6A, two-way ANOVA, main effect of genotype: *P*=0.0312; main effect of aphid infestation: *P*=0.168; effect of the interaction between genotype and aphid infestation: *P*=0.2140). The ethylene-induced marker gene *E4* was downregulated in *ACD* plants as compared with ‘UC82B’ (Supplementary Fig. S1 at *JXB* online), confirming that ethylene signalling was impaired in the *ACD* transgenic plants.

Since induction of *Pti5* by aphids did not require ethylene accumulation, we investigated the possible role of the alternative plant defensive hormones SA and JA. Transcript abundance of *Pti5* in response to aphid infestation was measured in the *NahG* tomato line, which expresses salicylate hydroxylase, an enzyme that diminishes SA accumulation by degrading SA to catechol (Gaffney *et al.*, 1993). Expression of *Pti5* was upregulated at 48 HAI in both the *NahG* and WT background tomato cultivar ‘MoneyMaker’ in response to aphid infestation (Fig. 6B; *P*=0.0277). No significant interaction between genotype and aphid treatment was observed (*P*=0.7489), indicating that induction of *Pti5* by aphids is also SA independent. However, constitutive transcript levels of *Pti5* were ~40 times higher in *NahG* as compared with ‘MoneyMaker’ (*P*<0.0001). We hypothesized that high constitutive *Pti5* expression in *NahG* plants resulted from the development of necrotic lesions in this genotype and not from a deficiency in SA. Several lines of evidence support our hypothesis: (i) constitutive expression of *Pti5* is higher in older than younger leaves, suggesting that expression of *Pti5* is regulated upon cell senescence (Gu *et al.*, 2000); (ii) *NahG* plants develop necrotic lesions through the vegetative growth stage (Brading *et al.*, 2000; Li *et al.*, 2002); (iii) expression of senescence-related genes in *NahG* plants has been related to the appearance of necrotic lesions (Avila *et al.*, 2013); and (iv) soil drenches of the SA analogue benzothiadiazole induce *Pti5* transcripts in tomato, which argues against the idea that SA is a negative regulator of this gene (Harel *et al.*, 2014).

We also measured transcript abundance of *Pti5* in the JA-deficient mutant tomato lines *acx1* and *spr2*. The *acx1* mutation in tomato blocks the first β-oxidation step of JA synthesis reducing JA content by 95% (Li *et al.*, 2005). The *spr2* line carries a loss-of-function mutation in *FATTY ACID DESATURASE 7* (*FAD7*) resulting in a 90% reduction in linolenic acid, a necessary precursor for JA synthesis (Li *et al.*, 2003). Both mutations almost completely eliminate expression of the JA-responsive marker gene *PROTEINASE INHIBITOR II* (*PI-II*) (Howe and Ryan, 1999; Li *et al.*, 2003; Li *et al.*, 2005; Avila *et al.*, 2012). Transcript abundance of *Pti5* was induced in both JA mutants in response to aphids at 48 HAI when compared with the WT background tomato cultivar ‘Castlemart’ (Fig. 6C, D; *P*<0.05). Similarly, there was no significant interaction between genotype and aphid treatment in either mutant (*P*>0.10), indicating that accumulation of JA is not required for aphid induction of *Pti5* transcription. However, JA mutants showed a small decrease in constitutive *Pti5* transcription levels compared with WT plants, although statistical significance was only reached in the *acx1* mutant (*acx1* genotype main effect *P*=0.0390; *spr2* genotype main effect *P*=0.6736). Thus, JA may potentially promote basal levels of *Pti5* expression.

In summary, our results clearly demonstrated that ethylene, SA, and JA accumulation are not required for transcriptional upregulation of *Pti5* by aphid infestation on tomato. While ethylene, SA, and JA are not the primary signals that trigger *Pti5* expression in response to aphids, they may potentially fine-tune constitutive or inducible *Pti5* expression levels; moreover, these phytohormone pathways may interact with *Pti5* signalling downstream of *Pti5* transcription. In fact, *Mi*-mediated aphid resistance is known to require SA (Martinez de Ilarduya *et al.*, 2003); therefore, SA-dependent defences and *Pti5*-dependent defences may act synergistically.

## Discussion

VIGS of *Pti5* resulted in increased aphid infestations on tomato, indicating that this ERF contributes to plant defences against piercing/sucking insects. These results illustrated the overlap between plant responses to aphids and plant responses to microbes, since *Pti5* and other ERFs are known for their roles in plant–pathogen interactions (Licausi *et al.*, 2013). As mentioned previously, *Pti5* contributes to host plant resistance against the bacterial pathogen *Pseudomonas syringae* pv. *syringae* (Thara *et al.*, 1999; He *et al.*, 2001). Moreover, it is also transcriptionally upregulated by *Trichoderma harzianum*, a beneficial soil fungus that promotes induced systemic resistance against the grey mould fungus *Botrytis cinerea* and other pathogens (Harel *et al.*, 2014). The *Pti5* gene product may contribute to aphid resistance by upregulating expression of PR genes, as is seen in the defence response against *P. syringae* (Gu *et al.*, 2000). In addition, ERFs are known to play other diverse roles in plant stress responses, including transcriptional regulation of secondary metabolite synthesis (Shoji *et al.*, 2010), cell death (Ogata *et al.*, 2013), reactive oxygen species signalling (Sewelam *et al.*, 2013), adjustments in primary metabolism (Vogel *et al.*, 2014), and crosstalk among hormone signalling pathways (Van der Does *et al.*, 2013). In other words, the suppressive effects of *Pti5* on aphids may be due to activation of direct defences and/or enhancement of defensive signalling by this transcription factor.

**Fig. 7.**
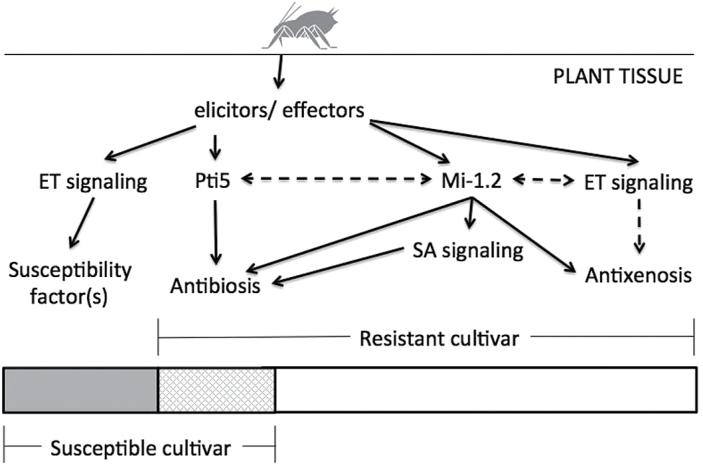
*Pti5*, *Mi-1.2*, and ethylene signalling in response to potato aphid feeding on tomato. Tomato plants recognize potato aphid secretions, which contain effectors that promote infestation, as well as elicitors that turn on defences. In the susceptible cultivar, ethylene signalling promotes aphid infestation by increasing aphid fecundity. In the resistant cultivar, *Mi-1.2* signalling reduces fecundity and increases mortality (antibiosis), and ethylene contributes to early stages of antixenotic defences. In both the susceptible and resistant cultivars, *Pti5* contributes to antibiosis. The shaded bar indicates the overlap between basal and R gene-mediated resistance. Arrows indicate steps that contribute to defence or susceptibility. Dashed arrows indicate unknown steps/mechanisms. ET, ethylene; SA, salicylic acid.

Silencing of *Pti5* resulted in increased aphid infestations on a genotype that lacks the *Mi-1.2* aphid resistance gene, which indicates that this gene contributes to basal levels of aphid resistance in susceptible hosts (Fig. 7). Suppression of *Pti5* expression also increased aphid population growth on a resistant (*Mi-1.2*+) cultivar, suggesting that this transcription factor might also contribute to R gene-mediated aphid resistance (Fig. 7). Previous studies have shown that transcriptional upregulation of *Pti5* in response to *Pseudomonas syringae* is enhanced when the pathogen is recognized by the R gene *Prf* (Thara *et al.*, 1999), and that *Pti5* contributes to *Prf/Pto*-mediated resistance against *P. syringae* through a direct physical interaction with the Pto kinase (Zhou *et al.*, 1997). However, transcriptional upregulation of *Pti5* by aphid infestation was similar in magnitude in genotypes with and without *Mi-1.2*, so this R gene does not appear to enhance transcription of *Pti5*. Upregulation of *Pti5* by aphids was also relatively slow and was not evident at 6h, whereas upregulation by *Pseudomonas syringae* is detectable as early as 1 HAI (Thara *et al.*, 1999). These differences suggest that *Pti5* might not play a direct role in *Mi-1.2*-dependent defences against aphids; instead, the effects of silencing *Pti5* on aphid numbers on the resistant (*Mi-1.2*+) cultivar might be due to additive effects between *Pti5*-dependent basal defences and R gene-mediated resistance. Alternatively, *Pti5* may participate in R gene-mediated aphid resistance, but enhancement of *Pti5* activity by *Mi-1.2* may rely on processes other than transcriptional activation. The activity of some ERFs is regulated by alternative splicing of the mRNA, post-translational modifications of the protein, and interactions with other transcription factors and structural proteins (Licausi *et al.*, 2013). Phosphorylation of the Pti5 protein by the Pto kinase enhances the DNA-binding activity of this transcription factor (Zhou *et al.*, 1997), and phosphorylation of ERF6 in *Arabidopsis* promotes its role in *B. cinerea* resistance (Meng *et al.*, 2013). Moreover, the susceptibility of ERF proteins to proteolysis and the affinity and specificity with which they bind DNA can be influenced by interacting proteins. For example, ORF1, an ERF in tobacco, interacts synergistically with basic helix–loop–helix transcription factors to promote expression of genes for nicotine synthesis (De Boer *et al.*, 2011). Thus, it is possible that *Mi-1.2* could impact post-transcriptional regulation of *Pti5* or the expression of interacting partners that modify *Pti5* activity.

Our results also indicated that ethylene is not required for transcriptional upregulation of *Pti5* by aphids, despite this gene’s classification as an ERF. Aphid infestation induces *Pti5* expression even in genotypes that express *ACD*, a transgene that inhibits ethylene synthesis and suppresses expression of *E4* and other ethylene-responsive genes. This is consistent with previous reports that *Pti5* is not induced by exogenous ethylene (Thara *et al.*, 1999; Gu *et al.*, 2000), and that *Pseudomonas syringae* is able to upregulate this gene in a mutant tomato line with impaired ethylene perception, the *Never-ripe* (*Nr*) mutant (Thara *et al.*, 1999). These findings highlight the fact that transcription factors are classified as ERFs based not on empirical evidence of ethylene responsiveness but on the presence of a conserved domain that can bind the GCC box, which is in turn common among genes that are upregulated by ethylene. In contrast to this classification scheme, a growing body of evidence has revealed that the ERF family does much more than mediate ethylene-responsive induction of GCC box-containing genes. For example, chromatin immunoprecipitation assays have shown that another ERF in tomato, Pti4, can bind to promoters that do not contain a GCC box, and microarray analysis has revealed that the majority of genes that were upregulated as a result of overexpression of *Pti4* lacked this motif (Chakravarthy *et al.*, 2003). The specificity of ERF binding can also vary in response to different stimuli; ERF1 in *Arabidopsis* binds to the GCC box in response to biotic stresses but interacts with the DRE/CRT motif when activated by abiotic stimuli (Cheng *et al.*, 2013). Furthermore, ERFs can mediate plant responses to hormones other than ethylene, including jasmonates, abscisic acid, and cytokinins (Licausi *et al.*, 2013).

While upstream ethylene signalling is not required for *Pti5* induction, it has been shown previously that ERFs can act as a point of convergence between ethylene and other signalling pathways (Lorenzo *et al.*, 2003). However, our results suggest that ethylene and *Pti5* play divergent rather than convergent roles in plant–aphid interactions (Fig. 7). In susceptible plants that lack *Mi-1.2*, the *Pti5* gene contributes to basal resistance, whereas ethylene signalling promotes aphid infestation. In the absence of *Mi-1.2*, we found that suppressing ethylene synthesis with the *ACD* transgene depressed aphid population growth; likewise, Mantelin *et al.* (2009) showed that inhibiting ethylene synthesis by silencing ACC synthases decreased aphid life spans. Furthermore, we observed higher levels of constitutive *Pti5* expression in the ethylene-deficient *ACD* plants than in WT controls, suggesting that ethylene suppresses basal *Pti5* expression. Similarly, application of the ethylene precursor ACC inhibited induction of the jasmonate-responsive ERFs at the *NIC2* locus in tobacco (Shoji *et al.*, 2010). Thus, it is possible for ethylene and ERFs to act antagonistically, and this appears to be the case for ethylene and *Pti5* in basal aphid resistance.

*Pti5* and ethylene also play distinctly different roles in tomato genotypes that carry the *Mi-1.2* aphid resistance gene. *Pti5* is induced sometime after 6h and contributes to antibiotic defences that limit aphid survival or reproduction on the host. In contrast, ethylene does not help or hinder *Mi-1.2*-mediated antibiosis, which is unaffected by the *ACD* transgene or AVG treatment (our results), the *Nr* mutation, or the pharmacological inhibitor 1-methylcyclopropene (Mantelin *et al.*, 2009). Instead, we found that ethylene contributes to *Mi-1.2*-mediated antixenotic defences that limit aphid settling on the host and that act early in the interaction (6h and earlier). These results illustrate that ethylene can play different roles in different genetic backgrounds, promoting infestation on compatible hosts but contributing to resistance in backgrounds that carry a relevant R gene. This is consistent with the fact that ethylene-associated transcripts are responsive to aphid feeding on both resistant (*Mi-1.2+)* and susceptible (*Mi-1.2*–) cultivars (Anstead *et al.*, 2010). In addition, our findings suggest that the antibioitic and antixenotic components of *Mi-1.2-*mediated aphid resistance are due to separate defence mechanisms regulated through different signalling pathways. Louis *et al.* (2012) also reported a divergence in molecular signalling between antibiotic and antixenotic components of basal aphid resistance in *Arabidopsis*. We have shown previously that, in addition to factors that inhibit ingestion from the phloem, *Mi*-*1.2*-mediated resistance also involves earlier-acting factors that deter sampling in the mesophyll or epidermis (Pallipparambil *et al.*, 2010). Therefore, it is possible that these resistance factors localized outside the vascular tissue are involved in ethylene-dependent antixenosis. These results advance our understanding of the mechanisms of R gene-mediated aphid resistance and also expand the potential applications of ERFs, overexpression of which has been shown previously to enhance pathogen resistance and abiotic stress tolerance in multiple plant species (Xu *et al.*, 2011).

## Supplementary data

Supplementary data are available at *JXB* online.

Supplementary Fig. S1. Expression of E4 ethylene-responsive gene in the *ACD* transgenic line.

Supplementary Data

## Abbreviations:

ACC: 1-aminocyclopropane-1-carboxylic acid
ACD: ACC deaminase
ANOVA: analysis of variance
AVG: aminoethoxyvinylglycine
DAI: days after infestation
EREBP: ethylene-responsive element binding protein
ERF: ethylene response factor
HAI: h after infestation
JA: jasmonic acid
PR: pathogenesis-related
R: resistance
RT-qPCR: real-time quantitative PCR
SA: salicylic acid
TRV: tobacco rattle virus
VIGS: virus-induced gene silencing
WT: wild type.
