# Supplementary material for: The ethylene response factor Pti5 contributes to potato aphid resistance in tomato independent of ethylene signalling
Source: J Exp Bot. 2014 Dec 11;66(2):559–70. doi: 10.1093/jxb/eru472 (PMC4286409; doi:10.1093/jxb/eru472)
Supplement: Supplementary Data [file supp_66_2_559__index.html]

The ethylene response factor Pti5 contributes to potato aphid resistance in tomato independent of ethylene signalling — The ethylene response factor Pti5 contributes to potato aphid resistance in tomato independent of ethylene signalling — Supplementary Data 

# The ethylene response factor Pti5 contributes to potato aphid resistance in tomato independent of ethylene signalling

## Supplementary Data

Data files

**Files in this Data Supplement:**

- Supplementary Data - Supplementary Data
