# Supplementary material for: The ethylene response factor Pti5 contributes to potato aphid resistance in tomato independent of ethylene signalling
Source: J Exp Bot. 2014 Dec 11;66(2):559–70. doi: 10.1093/jxb/eru472 (PMC4286409; doi:10.1093/jxb/eru472)
Supplement: Supplementary Data [file supp_eru472_jexbot134304_file001.pdf]

The ethylene response factor Pti5 contributes to potato aphid resistance in tomato independent of ethylene signaling

Chengjun Wu, Carlos A. Avila, and Fiona L. Goggin

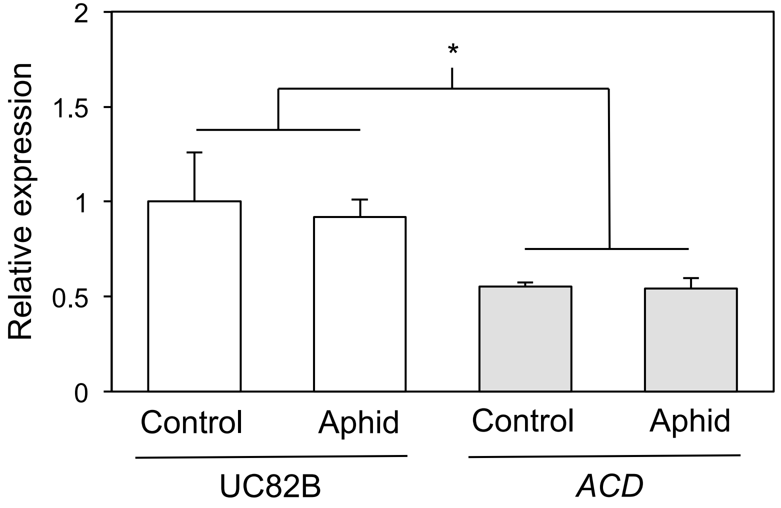

Supplemental Figure 1. Expression of E4 ethylene responsive gene in ACD transgenic line. Relative expression of *E4* was measured by RT-qPCR 48-hrs after potato aphid infestation in ethylene synthesis-deficient *ACD* tomato plants. Gene expression was normalized relative to Ribosomal Protein L2. Asterisk (*) denote statistically significant differences at α=0.05, and error bars represent SEM
